# (*Z*)-3-(4-Chloro­phen­yl)-2-{[*N*-(2-formyl­phen­yl)-4-methyl­benzene­sulfonamido]­meth­yl}prop-2-ene­nitrile

**DOI:** 10.1107/S160053681105241X

**Published:** 2011-12-14

**Authors:** R. Madhanraj, S. Murugavel, D. Kannan, M. Bakthadoss

**Affiliations:** a Department of Physics, Ranipettai Engineering College, Thenkadapathangal, Walaja 632 513, India; b Department of Physics, Thanthai Periyar Government Institute of Technology, Vellore 632 002, India; c Department of Organic Chemistry, University of Madras, Maraimalai Campus, Chennai 600 025, India

## Abstract

In the title compound, C_24_H_19_ClN_2_O_3_S, the sulfonyl-bound benzene ring forms dihedral angles of 38.1 (2) and 81.2 (1)°, respectively, with the formyl benzene and benzene rings. The mol­ecular conformation is stabilized by a weak intra­molecular C—H⋯O hydrogen bond, which generates an *S*(5) ring motif. The crystal packing is stabilized by C—H⋯O hydrogen bonds, which generate *C*(7) zigzag chains along [010] and *R*_3_^3^(19) ring motifs along [010]. The crystal packing is further stabilized by C—Cl⋯π inter­actions [Cl⋯centroid = 3.456 (2) Å and C—Cl⋯centroid = 173.4 (2)°].

## Related literature

For background to the pharmacological uses of sulfonamides, see: Korolkovas (1988 ▶); Mandell & Sande (1992 ▶). For related structures, see: Ranjith *et al.* (2009 ▶); Aziz-ur-Rehman *et al.* (2010 ▶). For hydrogen-bond motifs, see: Bernstein *et al.* (1995 ▶).
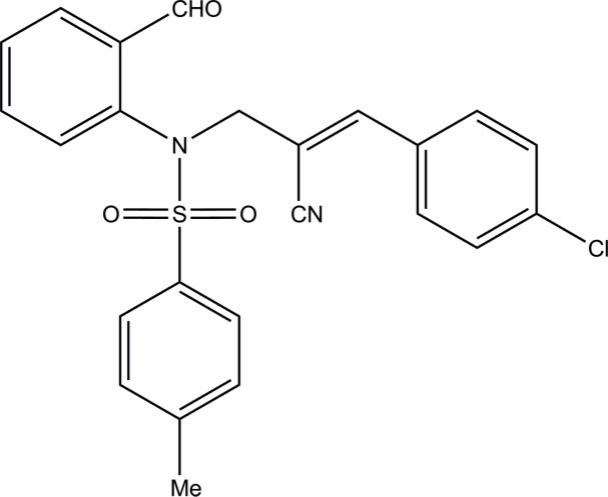


## Experimental

### 

#### Crystal data

C_24_H_19_ClN_2_O_3_S
*M* *_r_* = 450.92
Orthorhombic, 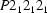

*a* = 8.9795 (5) Å
*b* = 10.1590 (5) Å
*c* = 25.1050 (13) Å
*V* = 2290.1 (2) Å^3^
*Z* = 4
Mo *K*α radiation
μ = 0.29 mm^−1^
*T* = 293 K
0.25 × 0.23 × 0.17 mm

#### Data collection

Bruker APEXII CCD diffractometer
Absorption correction: multi-scan (*SADABS*; Sheldrick, 1996 ▶) *T*_min_ = 0.931, *T*_max_ = 0.953
12721 measured reflections
4850 independent reflections
3396 reflections with *I* > 2σ(*I*)
*R*_int_ = 0.024

#### Refinement

*R*[*F* ^2^ > 2σ(*F* ^2^)] = 0.041
*wR*(*F* ^2^) = 0.111
*S* = 1.03
4850 reflections
281 parameters
H-atom parameters constrained
Δρ_max_ = 0.22 e Å^−3^
Δρ_min_ = −0.21 e Å^−3^
Absolute structure: Flack (1983 ▶), 2049 Friedel pairs
Flack parameter: 0.06 (8)

### 

Data collection: *APEX2* (Bruker, 2004 ▶); cell refinement: *APEX2* and *SAINT* (Bruker, 2004 ▶); data reduction: *SAINT* and *XPREP* (Bruker, 2004 ▶); program(s) used to solve structure: *SHELXS97* (Sheldrick, 2008 ▶); program(s) used to refine structure: *SHELXL97* (Sheldrick, 2008 ▶); molecular graphics: *ORTEP-3* (Farrugia, 1997 ▶; software used to prepare material for publication: *SHELXL97* and *PLATON* (Spek, 2009 ▶).

## Supplementary Material

Crystal structure: contains datablock(s) global, I. DOI: 10.1107/S160053681105241X/bt5740sup1.cif

Structure factors: contains datablock(s) I. DOI: 10.1107/S160053681105241X/bt5740Isup2.hkl

Supplementary material file. DOI: 10.1107/S160053681105241X/bt5740Isup3.cml

Additional supplementary materials:  crystallographic information; 3D view; checkCIF report

## Figures and Tables

**Table 1:** Hydrogen-bond geometry (Å, °)

*D*—H⋯*A*	*D*—H	H⋯*A*	*D*⋯*A*	*D*—H⋯*A*
C15—H15*A*⋯O3	0.97	2.43	2.890 (3)	109
C3—H3⋯O2^i^	0.93	2.57	3.345 (3)	141
C15—H15*A*⋯O2^ii^	0.97	2.57	3.385 (3)	142
C23—H23⋯O1^iii^	0.93	2.45	3.114 (4)	128

Symmetry codes: (i) 
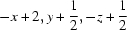
; (ii) 
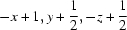
; (iii) 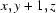.

**Fig. 1.**
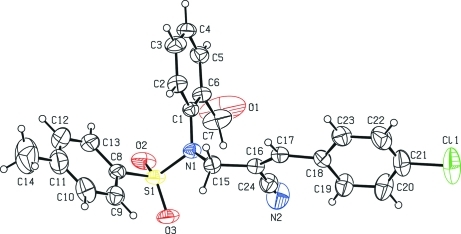
The molecular structure of the title compound with the atom numbering scheme. Displacement ellipsoids are drawn at the 30% probability level. H atoms are presented as a small cycles of arbitrary radius.

**Fig. 2.**
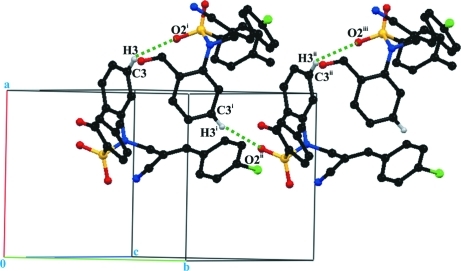
Part of the crystal structure of (I) showing C—H···O hydrogen bonds (dotted lines), with the formation of C(7) zig zag chains along [010]. [Symmetry codes: (i)2-x, 1/2+y, 1/2-z; (ii)x, 1+y, z; (iii) 2-x, 3/2+y, 1/2-z].

**Fig. 3.**
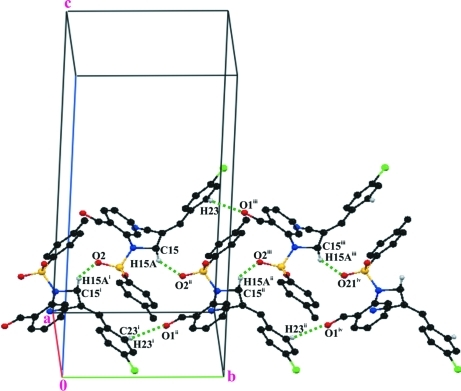
Part of the crystal structure of (I) showing C—H···O hydrogen bonds (dotted lines), with the formation of R33(19) ring motifs along [010] [Symmetry codes: (i)1-x, -1/2+y, 1/2-z; (ii)1-x, 1/2+y, 1/2-z; (iii)x, 1+y, z; (iv)1-x, 3/2+y, 1/2-z].

**Fig. 4.**
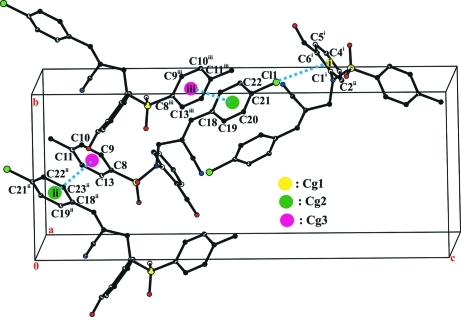
A view of the C—Cl···π and π—π interactions (dotted lines) in the crystal structure of the title compound. Cg1, Cg2 and Cg3 denotes centroids of the C1–C6 benzene ring, C8–C13 benzene ring and C18–C23 benzene ring, respectively. [Symmetry codes: (i)-1/2+x, 3/2-y, 1-z; (ii)1-x, -1/2+y, 1/2-z; (iii)1-x, 1/2+y, 1/2-z].

## supplementary crystallographic information

### Comment

Sulfonamide drugs are widely used for the treatment of certain infections caused by Gram-positive and Gram-negative microorganisms, some fungi, and certain protozoa (Korolkovas, 1988, Mandell & Sande, 1992). In view of this biological importance, the crystal structure of the title compound has been determined and the results are presented here.

Fig. 1. shows a displacement ellipsoid plot of (I), with the atom numbering scheme. The S1 atom shows a distorted tetrahedral geometry, with O2—S1—O3[119.8 (1)°] and N1—S1—C8[107.5 (1)°] angles deviating from ideal tetrahedral values. The sum of bond angles around N1 (352°) indicates that N1 is in *sp*^2^ hybridization. The sulfonyl bound phenyl (C8–C13) ring forms dihedral angles of 38.1 (2)° and 81.2 (2)°, respectively, with the formyl phenyl (C1–C6) and phenyl (C18—C23) rings. The dihedral angle between formyl phenyl and phenyl rings is 87.3 (1)°. The Cl1 atom deviates from the plane of attached ring by -0.031 (2) Å. The carbonitrile side chain (C16–C24–N2) is almost linear, with the angle around central carbon atom being 176.9 (3)°. The geometric parameters of the title molecule agrees well with those reported for similar structures (Ranjith *et al.,* 2009, Aziz-ur-Rehman *et al.,* 2010).

Ihe molecular structure is stabilized by C15—H15A···O3 intramolecular hydrogen bond, forming S(5) ring motif (Bernstein *et al.,* 1995) (Table 1). The crystal packing is stabilized by intermolecular C—H···O hydrogen bonds. The formation of the framework can be explained in terms of two-one substructures. In the first substructure, atom C3 in the molecule at (*x, y, z*) acts as a hydrogen bond donor to atom O2 in the molecule at (*2-x,1/2+y,1/2-z*) generating C(7) zig zag chains which are running along [010] (Fig. 2). In the second substructure, three molecules are linked by the combination of C15—H15A···O2 and C23—H23···O1 intermolecular hydrogen bonds generating *R_3_^3^*(19) ring motifs along [010] (Fig. 3). The crystal structure is further stabilized by C—Cl···.π interactions involving chlorine Cl1 and benzene ring (C1–C6), with Cl1···centroid(Cg^i^) distance of 3.456 (2) Å and C21—Cl1···Cg^i^ angle of 173.4 (2)° (Symmetry code as in Fig. 4). The crystal packing also exhibts π—π interactions with centroid—centroid distances: Cg2—Cg3^ii^ = 3.884 (2) Å and Cg3—Cg2^iii^ = 3.884 (2) Å (Fig. 4; Cg2 and Cg3 are the centroids of C18–C23 benzene ring and C8–C13 benzene ring, respectively, symmetry code as in Fig. 4).

### Experimental

A solution of *N*-(formylphenyl)(4-methylbenzene)sulfonamide (1 mmol, 0.28 g) and potassium carbonate (1.5 mmol, 0.21 g) in acetonitrile solvent was stirred for 15 minutes at room temperature. To this solution, (*E*)-2-(bromomethyl)-3-(4-chlorophenyl)prop-2-enenitrile (1.2 mmol, 0.30 g) was added dropwise till the addition is complete. After the completion of the reaction, as indicated by TLC, acetonitile was evaporated. Ethylacetate (15 ml) and water (15 ml) were added to the crude mass. The organic layer was dried over anhydrous sodium sulfate. Removal of solvent led to the crude product, which was purified through pad of silica gel (100–200 mesh) using ethylacetate and hexanes (1:9) as solvents. The pure title compound was obtained as a colourless solid (0.41 g, 90 % yield). Recrystallization was carried out using ethylacetate as solvent.

### Refinement

H atoms were positioned geometrically, with C—H = 0.93–0.98 Å and constrained to ride on their parent atom, with *U*_iso_(H)=1.5*U*_eq_ for methyl H atoms and 1.2*U*_eq_(C) for other H atoms.

### Crystal data

**Table:**

C_24_H_19_ClN_2_O_3_S	*F*(000) = 936
*M**_r_* = 450.92	*D*_x_ = 1.308 Mg m^−^^3^
Orthorhombic, *P*2_1_2_1_2_1_	Mo *K*α radiation, λ = 0.71073 Å
Hall symbol: P 2ac 2ab	Cell parameters from 4939 reflections
*a* = 8.9795 (5) Å	θ = 2.2–26.9°
*b* = 10.1590 (5) Å	µ = 0.29 mm^−^^1^
*c* = 25.1050 (13) Å	*T* = 293 K
*V* = 2290.1 (2) Å^3^	Block, yellow
*Z* = 4	0.25 × 0.23 × 0.17 mm

### Data collection

**Table:**

Bruker APEXII CCD diffractometer	4850 independent reflections
Radiation source: fine-focus sealed tube	3396 reflections with *I* > 2σ(*I*)
graphite	*R*_int_ = 0.024
Detector resolution: 10.0 pixels mm^-1^	θ_max_ = 26.9°, θ_min_ = 2.2°
ω scans	*h* = −11→11
Absorption correction: multi-scan (*SADABS*; Sheldrick, 1996)	*k* = −12→10
*T*_min_ = 0.931, *T*_max_ = 0.953	*l* = −31→31
12721 measured reflections	

### Refinement

**Table:**

Refinement on *F*^2^	Secondary atom site location: difference Fourier map
Least-squares matrix: full	Hydrogen site location: inferred from neighbouring sites
*R*[*F*^2^ > 2σ(*F*^2^)] = 0.041	H-atom parameters constrained
*wR*(*F*^2^) = 0.111	*w* = 1/[σ^2^(*F*_o_^2^) + (0.0572*P*)^2^ + 0.0529*P*] where *P* = (*F*_o_^2^ + 2*F*_c_^2^)/3
*S* = 1.03	(Δ/σ)_max_ < 0.001
4850 reflections	Δρ_max_ = 0.22 e Å^−^^3^
281 parameters	Δρ_min_ = −0.21 e Å^−^^3^
0 restraints	Absolute structure: Flack (1983), 2049 Friedel pairs
Primary atom site location: structure-invariant direct methods	Flack parameter: 0.06 (8)

### Special details

**Table:**

Geometry. All esds (except the esd in the dihedral angle between two l.s. planes) are estimated using the full covariance matrix. The cell esds are taken into account individually in the estimation of esds in distances, angles and torsion angles; correlations between esds in cell parameters are only used when they are defined by crystal symmetry. An approximate (isotropic) treatment of cell esds is used for estimating esds involving l.s. planes.
Refinement. Refinement of F^2^ against ALL reflections. The weighted R-factor wR and goodness of fit S are based on F^2^, conventional R-factors R are based on F, with F set to zero for negative F^2^. The threshold expression of F^2^ > 2sigma(F^2^) is used only for calculating R-factors(gt) etc. and is not relevant to the choice of reflections for refinement. R-factors based on F^2^ are statistically about twice as large as those based on F, and R- factors based on ALL data will be even larger.

### Fractional atomic coordinates and isotropic or equivalent isotropic displacement parameters (Å^2^)

**Table:**

	*x*	*y*	*z*	*U*_iso_*/*U*_eq_	
C11	0.7604 (6)	0.5498 (5)	0.07359 (14)	0.1160 (13)	
C14	0.8095 (7)	0.6089 (6)	0.02121 (16)	0.201 (3)	
H14A	0.7682	0.5588	−0.0077	0.301*	
H14B	0.9162	0.6071	0.0190	0.301*	
H14C	0.7754	0.6983	0.0190	0.301*	
O1	0.8121 (4)	0.1434 (4)	0.3700 (2)	0.219 (2)	
C1	0.8617 (3)	0.4264 (2)	0.28758 (8)	0.0479 (5)	
C2	0.9741 (3)	0.4987 (2)	0.26417 (10)	0.0643 (6)	
H2	0.9507	0.5693	0.2421	0.077*	
C3	1.1211 (3)	0.4665 (3)	0.27345 (12)	0.0768 (7)	
H3	1.1962	0.5159	0.2576	0.092*	
C4	1.1574 (3)	0.3637 (3)	0.30530 (11)	0.0717 (7)	
H4	1.2568	0.3427	0.3113	0.086*	
C5	1.0479 (3)	0.2920 (3)	0.32827 (10)	0.0666 (7)	
H5	1.0733	0.2215	0.3501	0.080*	
C6	0.8986 (3)	0.3211 (2)	0.32010 (9)	0.0582 (6)	
C7	0.7854 (4)	0.2419 (3)	0.34760 (14)	0.1041 (12)	
H7	0.6874	0.2715	0.3472	0.125*	
C8	0.6734 (3)	0.4380 (3)	0.16954 (10)	0.0628 (6)	
C9	0.5954 (3)	0.5421 (3)	0.14801 (11)	0.0779 (8)	
H9	0.5124	0.5756	0.1656	0.094*	
C10	0.6406 (5)	0.5966 (4)	0.10032 (13)	0.1040 (11)	
H10	0.5876	0.6671	0.0862	0.125*	
C12	0.8370 (4)	0.4450 (5)	0.09478 (17)	0.1209 (15)	
H12	0.9188	0.4113	0.0766	0.145*	
C13	0.7949 (3)	0.3886 (4)	0.14272 (13)	0.0928 (10)	
H13	0.8482	0.3181	0.1567	0.111*	
C15	0.6569 (3)	0.5935 (2)	0.28573 (9)	0.0611 (6)	
H15A	0.5668	0.6081	0.2653	0.073*	
H15B	0.7326	0.6532	0.2724	0.073*	
C16	0.6269 (3)	0.6244 (2)	0.34307 (8)	0.0537 (5)	
C17	0.6670 (3)	0.7373 (2)	0.36505 (9)	0.0560 (6)	
H17	0.7277	0.7897	0.3438	0.067*	
C18	0.6318 (3)	0.7938 (2)	0.41719 (9)	0.0570 (6)	
C19	0.5132 (3)	0.7545 (3)	0.44855 (11)	0.0837 (9)	
H19	0.4505	0.6878	0.4368	0.100*	
C20	0.4874 (5)	0.8130 (3)	0.49684 (12)	0.1036 (12)	
H20	0.4072	0.7859	0.5176	0.124*	
C21	0.5776 (5)	0.9097 (4)	0.51450 (12)	0.1022 (12)	
C22	0.6900 (5)	0.9554 (3)	0.48410 (14)	0.1018 (11)	
H22	0.7488	1.0250	0.4958	0.122*	
C23	0.7163 (3)	0.8974 (3)	0.43569 (12)	0.0770 (8)	
H23	0.7937	0.9291	0.4146	0.092*	
C24	0.5434 (3)	0.5271 (3)	0.37117 (11)	0.0716 (7)	
N1	0.7076 (2)	0.45611 (17)	0.27763 (8)	0.0551 (5)	
N2	0.4797 (4)	0.4447 (3)	0.39244 (12)	0.1108 (10)	
O2	0.6758 (2)	0.24175 (16)	0.23469 (9)	0.0834 (6)	
O3	0.46666 (17)	0.39646 (19)	0.23817 (8)	0.0807 (5)	
Cl1	0.5431 (2)	0.98128 (16)	0.57586 (4)	0.1878 (7)	
S1	0.62059 (6)	0.37202 (6)	0.23103 (3)	0.06219 (18)	

### Atomic displacement parameters (Å^2^)

**Table:**

	*U*^11^	*U*^22^	*U*^33^	*U*^12^	*U*^13^	*U*^23^
C11	0.119 (3)	0.147 (4)	0.081 (2)	−0.033 (3)	0.009 (2)	−0.028 (2)
C14	0.248 (6)	0.258 (7)	0.096 (3)	−0.088 (6)	0.045 (3)	0.002 (4)
O1	0.125 (2)	0.181 (3)	0.353 (5)	0.015 (2)	0.035 (3)	0.203 (4)
C1	0.0432 (12)	0.0425 (11)	0.0580 (11)	−0.0027 (10)	−0.0012 (10)	−0.0052 (10)
C2	0.0609 (16)	0.0558 (14)	0.0762 (15)	−0.0082 (12)	0.0038 (13)	0.0108 (12)
C3	0.0523 (15)	0.0728 (17)	0.1051 (19)	−0.0189 (15)	0.0065 (16)	−0.0079 (17)
C4	0.0430 (14)	0.0819 (19)	0.0902 (17)	0.0019 (15)	−0.0090 (13)	−0.0203 (16)
C5	0.0646 (17)	0.0626 (16)	0.0725 (15)	0.0150 (15)	−0.0119 (13)	−0.0006 (13)
C6	0.0498 (14)	0.0530 (14)	0.0716 (13)	0.0016 (11)	0.0002 (11)	0.0059 (11)
C7	0.077 (2)	0.088 (2)	0.148 (3)	0.0056 (18)	0.0180 (19)	0.062 (2)
C8	0.0458 (13)	0.0663 (17)	0.0762 (15)	−0.0001 (12)	−0.0051 (11)	−0.0263 (13)
C9	0.079 (2)	0.0769 (19)	0.0783 (17)	0.0068 (16)	−0.0054 (14)	−0.0150 (15)
C10	0.137 (3)	0.089 (2)	0.086 (2)	−0.007 (2)	−0.014 (2)	−0.0118 (18)
C12	0.085 (3)	0.178 (5)	0.101 (3)	−0.005 (3)	0.020 (2)	−0.050 (3)
C13	0.0632 (18)	0.120 (3)	0.095 (2)	0.014 (2)	−0.0043 (16)	−0.038 (2)
C15	0.0680 (16)	0.0525 (13)	0.0626 (13)	0.0185 (12)	−0.0040 (11)	−0.0006 (11)
C16	0.0478 (12)	0.0512 (13)	0.0623 (12)	−0.0005 (13)	−0.0036 (11)	0.0016 (11)
C17	0.0517 (14)	0.0558 (14)	0.0604 (13)	−0.0009 (11)	−0.0002 (10)	0.0082 (12)
C18	0.0637 (15)	0.0499 (13)	0.0574 (12)	0.0003 (13)	−0.0082 (12)	0.0057 (10)
C19	0.100 (2)	0.074 (2)	0.0773 (17)	−0.0220 (18)	0.0195 (16)	−0.0119 (14)
C20	0.135 (3)	0.093 (2)	0.082 (2)	−0.012 (2)	0.037 (2)	−0.0069 (18)
C21	0.146 (4)	0.092 (3)	0.0687 (17)	0.012 (2)	−0.005 (2)	−0.0209 (17)
C22	0.119 (3)	0.082 (2)	0.105 (2)	−0.006 (2)	−0.021 (2)	−0.034 (2)
C23	0.0789 (18)	0.0617 (16)	0.0902 (19)	−0.0080 (15)	−0.0028 (15)	−0.0113 (15)
C24	0.0772 (18)	0.0632 (17)	0.0744 (15)	−0.0074 (16)	0.0024 (14)	−0.0077 (14)
N1	0.0479 (11)	0.0448 (10)	0.0727 (12)	0.0070 (9)	−0.0031 (9)	−0.0042 (9)
N2	0.134 (3)	0.0835 (19)	0.115 (2)	−0.0344 (19)	0.0280 (18)	−0.0016 (16)
O2	0.0650 (11)	0.0475 (10)	0.1378 (15)	−0.0014 (8)	−0.0240 (11)	−0.0125 (10)
O3	0.0386 (9)	0.0940 (14)	0.1095 (13)	−0.0011 (10)	0.0003 (9)	−0.0049 (11)
Cl1	0.2742 (18)	0.1942 (13)	0.0951 (7)	0.0109 (14)	0.0130 (9)	−0.0698 (8)
S1	0.0394 (3)	0.0540 (3)	0.0931 (4)	−0.0008 (3)	−0.0067 (3)	−0.0100 (3)

### Geometric parameters (Å, °)

**Table:**

C11—C10	1.354 (5)	C12—C13	1.386 (5)
C11—C12	1.375 (6)	C12—H12	0.9300
C11—C14	1.511 (6)	C13—H13	0.9300
C14—H14A	0.9600	C15—N1	1.482 (3)
C14—H14B	0.9600	C15—C16	1.498 (3)
C14—H14C	0.9600	C15—H15A	0.9700
O1—C7	1.173 (4)	C15—H15B	0.9700
C1—C2	1.379 (3)	C16—C17	1.323 (3)
C1—C6	1.386 (3)	C16—C24	1.427 (4)
C1—N1	1.438 (3)	C17—C18	1.464 (3)
C2—C3	1.380 (4)	C17—H17	0.9300
C2—H2	0.9300	C18—C23	1.378 (4)
C3—C4	1.355 (4)	C18—C19	1.383 (4)
C3—H3	0.9300	C19—C20	1.370 (4)
C4—C5	1.353 (4)	C19—H19	0.9300
C4—H4	0.9300	C20—C21	1.348 (5)
C5—C6	1.388 (3)	C20—H20	0.9300
C5—H5	0.9300	C21—C22	1.348 (5)
C6—C7	1.468 (4)	C21—Cl1	1.731 (3)
C7—H7	0.9300	C22—C23	1.371 (4)
C8—C13	1.377 (4)	C22—H22	0.9300
C8—C9	1.379 (4)	C23—H23	0.9300
C8—S1	1.748 (3)	C24—N2	1.146 (4)
C9—C10	1.380 (4)	N1—S1	1.6459 (19)
C9—H9	0.9300	O2—S1	1.4161 (18)
C10—H10	0.9300	O3—S1	1.4157 (17)
			
C10—C11—C12	118.5 (4)	C8—C13—H13	120.3
C10—C11—C14	121.6 (5)	C12—C13—H13	120.3
C12—C11—C14	119.9 (5)	N1—C15—C16	112.58 (19)
C11—C14—H14A	109.5	N1—C15—H15A	109.1
C11—C14—H14B	109.5	C16—C15—H15A	109.1
H14A—C14—H14B	109.5	N1—C15—H15B	109.1
C11—C14—H14C	109.5	C16—C15—H15B	109.1
H14A—C14—H14C	109.5	H15A—C15—H15B	107.8
H14B—C14—H14C	109.5	C17—C16—C24	122.5 (2)
C2—C1—C6	119.2 (2)	C17—C16—C15	122.2 (2)
C2—C1—N1	121.2 (2)	C24—C16—C15	115.1 (2)
C6—C1—N1	119.6 (2)	C16—C17—C18	130.9 (2)
C1—C2—C3	120.1 (2)	C16—C17—H17	114.6
C1—C2—H2	120.0	C18—C17—H17	114.6
C3—C2—H2	120.0	C23—C18—C19	116.9 (2)
C4—C3—C2	120.9 (2)	C23—C18—C17	118.8 (2)
C4—C3—H3	119.6	C19—C18—C17	124.2 (2)
C2—C3—H3	119.6	C20—C19—C18	120.6 (3)
C5—C4—C3	119.4 (2)	C20—C19—H19	119.7
C5—C4—H4	120.3	C18—C19—H19	119.7
C3—C4—H4	120.3	C21—C20—C19	120.3 (3)
C4—C5—C6	121.7 (2)	C21—C20—H20	119.8
C4—C5—H5	119.2	C19—C20—H20	119.8
C6—C5—H5	119.2	C20—C21—C22	121.0 (3)
C1—C6—C5	118.8 (2)	C20—C21—Cl1	119.4 (3)
C1—C6—C7	122.3 (2)	C22—C21—Cl1	119.5 (3)
C5—C6—C7	118.9 (2)	C21—C22—C23	118.9 (3)
O1—C7—C6	123.5 (3)	C21—C22—H22	120.6
O1—C7—H7	118.3	C23—C22—H22	120.6
C6—C7—H7	118.3	C22—C23—C18	122.1 (3)
C13—C8—C9	119.4 (3)	C22—C23—H23	118.9
C13—C8—S1	120.5 (2)	C18—C23—H23	118.9
C9—C8—S1	120.1 (2)	N2—C24—C16	176.9 (3)
C8—C9—C10	119.9 (3)	C1—N1—C15	117.97 (18)
C8—C9—H9	120.1	C1—N1—S1	118.12 (14)
C10—C9—H9	120.1	C15—N1—S1	116.15 (15)
C11—C10—C9	121.5 (4)	O3—S1—O2	119.82 (12)
C11—C10—H10	119.2	O3—S1—N1	106.40 (11)
C9—C10—H10	119.2	O2—S1—N1	105.83 (10)
C11—C12—C13	121.3 (4)	O3—S1—C8	108.02 (12)
C11—C12—H12	119.3	O2—S1—C8	108.70 (13)
C13—C12—H12	119.3	N1—S1—C8	107.46 (11)
C8—C13—C12	119.4 (4)		
			
C6—C1—C2—C3	−0.4 (4)	C17—C18—C19—C20	−179.6 (3)
N1—C1—C2—C3	−178.6 (2)	C18—C19—C20—C21	−0.1 (5)
C1—C2—C3—C4	0.2 (4)	C19—C20—C21—C22	3.4 (6)
C2—C3—C4—C5	0.0 (4)	C19—C20—C21—Cl1	−179.6 (3)
C3—C4—C5—C6	−0.1 (4)	C20—C21—C22—C23	−3.2 (6)
C2—C1—C6—C5	0.3 (3)	Cl1—C21—C22—C23	179.8 (3)
N1—C1—C6—C5	178.5 (2)	C21—C22—C23—C18	−0.2 (5)
C2—C1—C6—C7	178.2 (3)	C19—C18—C23—C22	3.3 (4)
N1—C1—C6—C7	−3.5 (4)	C17—C18—C23—C22	180.0 (3)
C4—C5—C6—C1	−0.1 (4)	C17—C16—C24—N2	−147 (6)
C4—C5—C6—C7	−178.1 (3)	C15—C16—C24—N2	37 (6)
C1—C6—C7—O1	170.6 (4)	C2—C1—N1—C15	−52.3 (3)
C5—C6—C7—O1	−11.5 (6)	C6—C1—N1—C15	129.5 (2)
C13—C8—C9—C10	−0.7 (4)	C2—C1—N1—S1	95.8 (2)
S1—C8—C9—C10	177.8 (2)	C6—C1—N1—S1	−82.4 (2)
C12—C11—C10—C9	0.4 (6)	C16—C15—N1—C1	−80.8 (3)
C14—C11—C10—C9	179.6 (4)	C16—C15—N1—S1	130.40 (19)
C8—C9—C10—C11	0.3 (5)	C1—N1—S1—O3	166.06 (17)
C10—C11—C12—C13	−0.8 (6)	C15—N1—S1—O3	−45.2 (2)
C14—C11—C12—C13	180.0 (4)	C1—N1—S1—O2	37.58 (19)
C9—C8—C13—C12	0.4 (4)	C15—N1—S1—O2	−173.70 (18)
S1—C8—C13—C12	−178.2 (3)	C1—N1—S1—C8	−78.43 (18)
C11—C12—C13—C8	0.4 (5)	C15—N1—S1—C8	70.29 (19)
N1—C15—C16—C17	138.6 (2)	C13—C8—S1—O3	−155.0 (2)
N1—C15—C16—C24	−45.5 (3)	C9—C8—S1—O3	26.5 (2)
C24—C16—C17—C18	−4.9 (4)	C13—C8—S1—O2	−23.5 (2)
C15—C16—C17—C18	170.8 (2)	C9—C8—S1—O2	157.92 (19)
C16—C17—C18—C23	164.1 (2)	C13—C8—S1—N1	90.6 (2)
C16—C17—C18—C19	−19.4 (4)	C9—C8—S1—N1	−88.0 (2)
C23—C18—C19—C20	−3.1 (4)		

### Hydrogen-bond geometry (Å, °)

**Table:**

*D*—H···*A*	*D*—H	H···*A*	*D*···*A*	*D*—H···*A*
C15—H15A···O3	0.97	2.43	2.890 (3)	109
C3—H3···O2^i^	0.93	2.57	3.345 (3)	141.
C15—H15A···O2^ii^	0.97	2.57	3.385 (3)	142.
C23—H23···O1^iii^	0.93	2.45	3.114 (4)	128.

Symmetry codes:  (i) −*x*+2, *y*+1/2, −*z*+1/2; (ii) −*x*+1, *y*+1/2, −*z*+1/2; (iii) *x*, *y*+1, *z*.
